# Anesthetic management of cesarean section in a patient with Takayasu’s arteritis: a case report

**DOI:** 10.1186/s40981-021-00494-0

**Published:** 2022-01-05

**Authors:** Taichi Ando, Makoto Sumie, Shoichi Sasaki, Miho Yoshimura, Keiko Nobukuni, Jun Maki, Katsuyuki Matsushita, Kazuhiro Shirozu, Midoriko Higashi, Ken Yamaura

**Affiliations:** 1 Department of Anesthesiology and Critical Care Medicine, Kyushu University Hospital, 3-1-1 Maidashi, Higashi-ku, Fukuoka, 812-8582 Japan; 2 Intensive Care Unit, Kyushu University Hospital, 3-1-1 Maidashi, Higashi-ku, Fukuoka, 812-8582 Japan; 3 Operating Rooms, Kyushu University Hospital, 3-1-1 Maidashi, Higashi-ku, Fukuoka, 812-8582 Japan

**Keywords:** Cesarean section, Spinal anesthesia, Takayasu’s arteritis

## Abstract

**Background:**

Takayasu’s arteritis (TA) is a chronic, progressive, inflammatory arteritis. We presented the case of cesarean section in a patient with TA.

**Case presentation:**

A 31-year-old pregnant woman with TA underwent a planned cesarean section at 34 weeks of pregnancy. She had stenosis of the cerebral and coronary arteries and heart failure due to aortic regurgitation. Spinal anesthesia was performed. In addition to standard monitoring, arterial blood pressure in the dorsalis pedis artery and regional cerebral tissue oxygen saturation were monitored. Intraoperative arterial blood pressure was maintained using continuous infusion of noradrenaline with a careful intermittent bolus infusion of phenylephrine. All the procedures were successfully performed without significant complications.

**Conclusions:**

In a pregnant woman with TA, severe stenosis of the cerebral and coronary arteries, and heart failure due to valvular heart disease, careful anesthetic management by selecting catecholamines and assessing the perfusion pressure for critical organs is important.

## Background

Takayasu’s arteritis (TA) is a disease characterized by non-specific inflammation of the aorta and its main branches [1]. The incidence of TA is estimated to be 1.2–2.6 per million, and it is common in the Asian population. TA commonly occurs in adolescents and young adult women [2]. Several studies on the anesthetic management of cesarean sections with TA have been reported. However, there are no reports of anesthetic management with spinal anesthesia alone for a cesarean section of patients with cervical vascular lesions, severe aortic regurgitation, and coronary artery stenosis caused by TA (Table 1) [3–9]. We report a successful anesthetic management of cesarean section with spinal anesthesia in a patient with stenosis in the branches of the aortic arch and coronary arteries and aortic regurgitation caused by TA.

**Table 1:** Past anesthetic management of cesarean section in patients with Takayasu’s arteritis

	Heart and vascular lesions	Anesthesia method	Monitoring
This case	Left CAA stenosis Bilateral VA stenosis Bilateral SA stenosis AR	Spinal anesthesia	Standard monitoring Arterial blood pressure (right dorsalis pedis artery) rSO_2_ FloTrac™
Dutta et al. [3]	No heart and vascular lesions	Spinal anesthesia	Standard monitoring Arterial blood pressure (right dorsalis pedis artery)
Gautam et al. [4]	Right CAA stenosis Left CAA stenosis MR AR	Epidural anesthesia	Standard monitoring Arterial blood pressure (right radial artery)
Lee et al. [5]	Left CCA occlusion Left SA occlusion DA aneurysm Left CA aneurysm Right CA stenosis	Epidural anesthesia	Standard monitoring Arterial blood pressure (right radial artery) rSO_2_ FloTrac™
Xiao et al. [6]	ICA stenosis SA stenosis RA stenosis MR	Epidural anesthesia	Standard monitoring Arterial blood pressure (femoral artery) rSO_2_
Kassa et al. [7]	No heart and vascular lesions	Spinal anesthesia	Standard monitoring
Varghese et al. [8]	Left CAA stenosis Left SA stenosis	CSEA	Standard monitoring
Gupta et al. [9]	Bilateral SA stenosis	CSEA	Standard monitoring

*AR* aortic regurgitation, *CAA* coronary artery aneurysm, *CAAS* common carotid artery stenosis, *CAS* coronary artery stenosis, *CSEA* combined spinal-epidural anesthesia, *DAA* descending aortic aneurysm, *MR* mitral regurgitation, *RAS* renal artery stenosis, *rSO*_*2*_ regional cerebral tissue oxygen saturation, *SAS* subclavian artery stenosis, *VAS* vertebral artery stenosis

## Case presentation

The patient in this case report was a 31-year-old woman (height, 160 cm; weight, 63 kg [57 kg when she was not pregnant]). She was diagnosed with TA at the age of 23 years and started treatment with steroids and immunosuppressive drugs. She had a spontaneous pregnancy and was admitted to our hospital at 23 weeks of pregnancy because of cardiovascular disease associated with TA. Her gravidity was two times, including artificial abortion at 18 years, and she was nulliparity. After admission to our hospital, daily vital signs, weight measurement, and cardiac ultrasonography once every 2 weeks were performed. As the pregnancy progressed, some signs of cardiac failure, such as postprandial chest tightness, cardiac enlargement, and pericardial effusion, became apparent. A cesarean section was planned at 34 weeks and 1 day of pregnancy, considering the risk of increased systemic vascular resistance and exacerbation of heart failure by straining, hyperventilation, and labor induction.

She had a medical history of bare-metal stent placement for a stenotic lesion in the left main trunk of the coronary artery at the age of 23 years and drug-eluting stent placement for a stenotic lesion in the circumflex branch at the age of 24 years. She was undergoing aspirin and clopidogrel antithrombotic therapy after drug-eluting stent implantation; however, clopidogrel was discontinued after 23 weeks of pregnancy in preparation for delivery, and antithrombotic therapy with aspirin alone was continued. She was also undergoing nifedipine and carvedilol therapy for hypertension.

The patient’s pulse in the bilateral radial arteries was not palpable due to inflammatory changes in the arteries associated with TA; however, the bilateral dorsalis pedis arteries were palpable. Blood pressure could not be measured in the upper arm. Her blood pressure was 131/65 mmHg, measured on the lower legs, and her pulse rate was 82 beats/min on admission. Her oxygen saturation levels using a pulse oximeter were available and 99% in fingers of both hands.

**Fig. 1:**
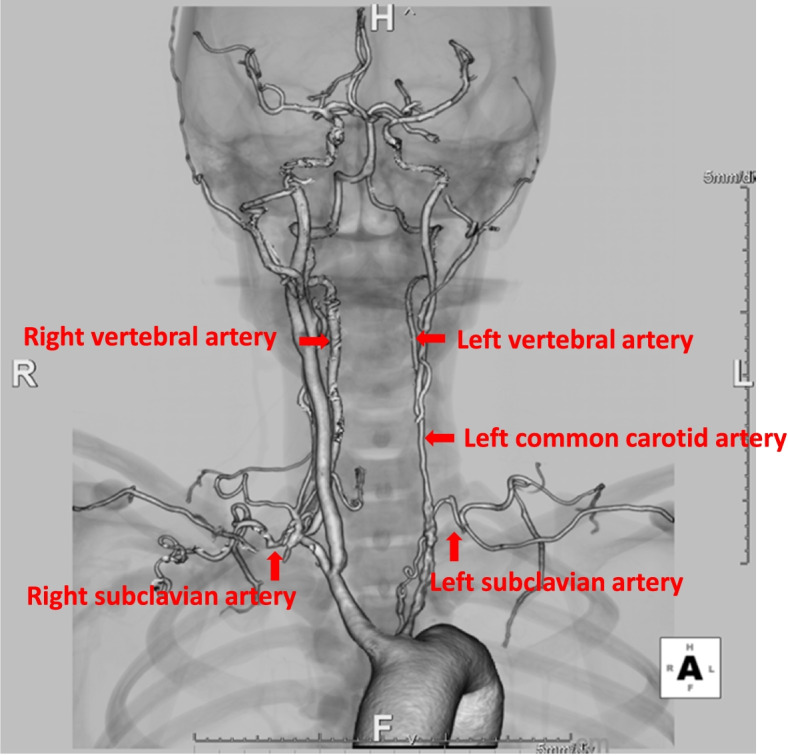
Three-dimensional computed tomography. Stenosis of the left common carotid artery, bilateral vertebral arteries, and bilateral subclavian arteries is shown (directional marker)

Imaging examinations, including head computed tomography, head magnetic resonance imaging, and brain single-photon emission computed tomography, revealed severe stenosis of the left common carotid artery, bilateral vertebral arteries, and bilateral subclavian arteries, with preserved blood flow from the right common carotid artery to the entire brain via the circle of Willis arteries (Fig. 1). Transthoracic echocardiography revealed aortic regurgitation, with a pressure half time of 309.7 ms, effective regurgitant valve opening area of 0.24 cm^2^, sinus of Valsalva diameter of 41.9 mm, and ascending aortic diameter of 40.7 mm. These findings indicated severe aortic regurgitation associated with aortic root dilatation.

Cesarean section was planned under spinal anesthesia. In addition to standard monitoring, we monitored arterial pressure, cardiac output at the dorsalis pedis artery using the FloTrac™ sensor (Edwards Lifesciences), and regional cerebral tissue oxygen saturation (rSO_2_) using the INVOS™ 5100C system (Covidien Japan K.K.).

**Fig. 2:**
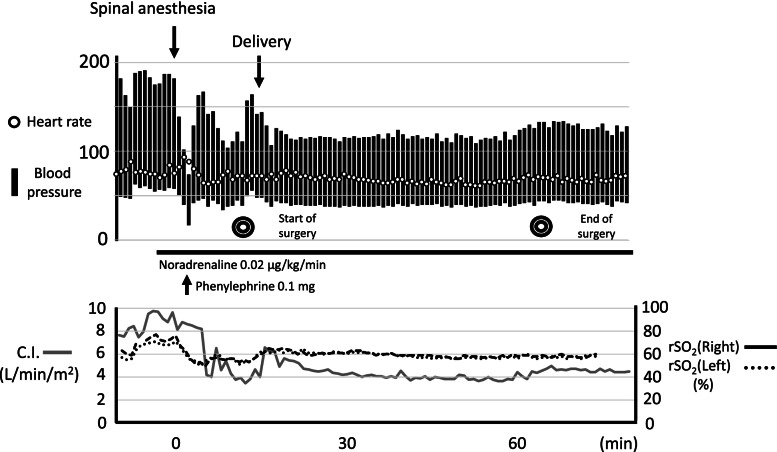
Intraoperative changes in circulatory dynamics and regional cerebral tissue oxygen saturation. rSO_2_, regional cerebral tissue oxygen saturation; C.I., cardiac index

A continuous dose of nicorandil was initiated after admission at 2 mg/h, followed by a continuous dose of noradrenaline at 0.02 μg/kg/min. Spinal anesthesia was performed with a 26-G pencil-point needle from the third and fourth lumbar intervertebral spaces, and 1.8 mL of 0.5% hyperbaric bupivacaine was administered with 10 μg of fentanyl and 100 μg of morphine. Immediately after the regional anesthetics administration, the systolic blood pressure decreased to 73 mmHg, and the mean blood pressure was 42 mmHg. rSO_2_ temporarily decreased from 56 to 50% on the right and from 63 to 52% on the left; however, there were no abnormal neurological findings. A single dose of 0.1 mg of phenylephrine was administered, and the patient’s blood pressure improved rapidly. Intraoperatively, the systolic blood pressure was maintained at 110–120 mmHg, the mean blood pressure was 60–70 mmHg, and the heart rate was 70–80 beats/min. After 10 min of regional anesthetics administration, a loss of cold sensation occurred in the Th5–S dermatomes. The infant was delivered 3 min after the initiation of surgery. Intravenous 10 units of oxytocin were administered immediately after placental delivery. Favorable uterine contractions were obtained; however, the patient’s blood pressure and heart rate did not change. The infant had Apgar scores of 8 and 9 points at 1 and 5 min, respectively, and weighed 1888 g. The umbilical artery blood pH was 7.364, pCO_2_ was 38.3 mmHg, base excess was − 3.2 mmol/L, and lactate was 2.8 mmol/L. The operative time was 57 min; blood loss was 1470 mL, including amniotic fluid; and the volume of fluid infusion was 693 mL of extracellular fluid preparation and 750 mL of hydroxyethyl starch preparation (Fig. 2). The postoperative course was uneventful; signs of cardiac failure are chest tightness, cardiac enlargement, and pericardial effusion disappeared, and both the mother and child were discharged without any complications on the 11th postoperative day.

## Discussion

In this case, stenosis of the left common carotid artery, bilateral vertebral artery, and bilateral subclavian artery was observed. Moreover, the patient had a history of treatment for coronary artery stenosis, and severe aortic regurgitation and pericardial effusion were observed. Therefore, we believed it was important to select the correct anesthetic method, to maintain perioperative cerebral blood flow, to maintain coronary artery blood flow, and to manage heart failure.

First, regarding the selection of anesthetic method, all the reports so far have reported on epidural anesthesia, spinal anesthesia, or a combination of both [3–9]. In previous reports, epidural anesthesia was selected to prevent sudden blood pressure deterioration [4–6]. In this case, the patient was under antiplatelet drug therapy. Therefore, general anesthesia was also an option because of concerns about bleeding complications associated with regional anesthesia. However, we selected regional anesthesia because of the possibility that airway edema was progressing due to pregnancy, heart failure, and severe tachycardia and hypertension due to insufficient depth of rapid sequence induction followed by myocardial oxygen supply-demand imbalance causing myocardial ischemia and exacerbation of heart failure. Furthermore, general anesthesia conceals neurological deficits, such as consciousness disturbance and paralysis due to cerebral ischemia. Epidural anesthesia is desirable to avoid sudden circulatory fluctuations, as per previous reports [4–6]. However, we selected spinal anesthesia because the patient was under aspirin therapy and the puncture needle was thinner than the epidural needle, and a sufficient anesthesia area could be obtained by a single puncture and drug administration and we could prepare effective vasopressors, even though epidural anesthesia is relatively safe without concern about spinal hematoma according to the guideline of regional anesthesia in patients receiving antithrombotic or thrombolytic therapy [10]. When spinal anesthesia was performed, we ensured to prevent hemodynamic deterioration caused by a rapid decrease in blood pressure and aggravation of aortic regurgitation associated with bradycardia.

For the treatment of hypotension during cesarean section under spinal anesthesia, phenylephrine tends to cause maternal bradycardia, whereas noradrenaline is not likely to cause bradycardia [11, 12]. A continuous administration of noradrenaline can prevent hypotension after spinal anesthesia during cesarean section and phenylephrine-induced bradycardia and reactive hypertension [13]. In contrast, noradrenaline increases myocardial contractility and heart rate by beta 1 receptor stimulation, which can lead to tachycardia [14]. In this case, to maintain circulation with spinal anesthesia, bradycardia and increased cardiac afterload should be prevented considering severe aortic regurgitation; however, tachycardia should also be prevented considering coronary artery disease. Therefore, continuous administration of low-dose noradrenaline was selected to prevent an excessive decrease in systemic vascular resistance and to maintain heart rate. Until the onset of these effects, a single dose of noradrenaline, which may cause tachycardia, was not selected, and a single dose of phenylephrine (0.1 mg) was carefully and appropriately administered to maintain blood pressure. The sufficient effect of the continuous administration of noradrenaline, which led to stable and appropriate blood pressure and heart rate, appeared at the beginning of the surgery. A previous study reported that noradrenaline was effective for various maternal and fetal outcomes of cesarean section with regional anesthesia to prevent low blood pressure [15]. Noradrenaline is not inferior to phenylephrine for fetal acidosis [16]. In this case, noradrenaline was effective for maintaining blood pressure during surgery, and both arterial blood pH of the umbilical cord and the infant’s Apgar score were also acceptable. To maintain coronary blood flow, we maintained sufficient diastolic blood pressure and preload and administered a continuous infusion of nicorandil. Lee et al. used cardiac output measured by the FloTrac™ sensor to indicate adequate fluid volume. However, they reported that the reliability was low due to the presence of vascular lesions [5]. In this case, we used the FloTrac™ sensor, and the cardiac index remained at greater than 3.5 L/min/m^2^; however, we recommend to carefully evaluate the result depending on which arterial was used for measurement.

Finally, there are several reports wherein invasive arterial blood pressure measurement and rSO_2_ monitoring were performed for patients with cervical vascular stenosis or obstruction and valvular disease as monitored in our case [4–6]. Lee et al. reported that rSO_2_ monitoring was useful for detecting transient decrease of cerebral perfusion in a patient who developed dysarthria, tinnitus, and stiff neck during cesarean section [5]. In our case, temporal decrease in rSO_2_ was observed in accordance with a decrease of blood pressure immediately after spinal anesthesia. Although neurological abnormalities were not observed, rSO_2_ may be monitored for cerebral blood flow assessment in pregnant women with TA.

## Conclusions

In anesthetic management of cesarean section in a parturient with TA, anesthesiologists should carefully select the anesthetic method and drug administration while considering oral administration of antiplatelet drugs, heart disease, and maintenance of organ blood flow associated with vascular lesions, followed by the measurement of blood pressure and monitoring.

## Data Availability

Not applicable.

## Abbreviations

pH: Potential of hydrogen
pCO_2_: Partial pressure of carbon dioxide
rSO_2_: Regional cerebral tissue oxygen saturation
TA: Takayasu’s arteritis
